# The Infected Air from Small-Pox Hospitals

**Published:** 1901-03-23

**Authors:** 


### The Institutional Workshop.

### THE INFECTED AIR FROM SMALL-POX HOSPITALS.

### AN ACCOUNT OF SOME METHODS OF STERILISING IT.

The general feeling is that when a patient suffering from small-pox has been removed to the hospital and the house where he lived has been disinfected all danger of infection is removed. It is apt to be forgotten that the hospital itself may be a source of infection if the air that escapes from it is not disinfected before reaching the outer air. Generally, isolation hospitals are built at some distance from ordinary habitations, but in large towns this is not altogether practicable, and it therefore becomes a matter of some importance to know how far the exit air from these hospitals can be sterilised so as to make it innocuous to those in the neighbourhood. To this end the late Dr. F. W. Barry prepared a memorandum for the Local Government Board showing what efforts had been made to sterilise the air from small-pox hospitals, and with what result.

Special attempts to attain this end had been made by the sanitary authorities of Barnsley, Nottingham, and Bradford, when Dr. Barry made his experiments. The aim was in each instance to withdraw the air from the small-pox wards along certain definite channels, and the subsequent sterilisation of this air by heat, in accordance with the suggestions made by Professor Burdon Sanderson in his evidence given before the Royal Commission respecting Small-pox and Fever Hospitals. Dr. Barry accordingly examined the arrangements in the small-pox wards in these places.

In the Kendray Hospital at Barnsley the small-pox hospital is circular in shape, with two projections, one of which contains the nurses' rooms, and the other slop-closets. The circular portion of the building is divided into two chambers by means of a circular wall erected at a distance of 18 feet from the outer wall. The outer space forms the hospital proper, divided into two wards, a male and a female, the inner space enclosed by it contains, in the basement, the furnace in connection with the apparatus for warming the wards, and upon an upper floor the apparatus for sterilising the exit air. The wards are ventilated in three ways. First there are four openings in the outer wall of the ward at the floor level: these consist of gratings 13½ inches long by 8½ inches wide, of which space about half is available. A second series of three gratings, each about 20½ inches by 3¾ inches in size, open over the hot-water coils by which the wards are warmed, and are intended to act as air inlets in cold weather. In each ward there are seven windows, and of these the upper sash of four of them can be opened. All these are meant to act as inlets only. The air outlets are situated in the inner circular wall near the ceiling, and each communicating directly with a Keeling's sewer-gas destructor, where the air coming out of the wards is subjected to the flame of an Argand gas-burner.

The first experiment made by Dr. Barry was intended to see if the extractive power provided was sufficient to prevent the infected air escaping from the wards by any other outlets than those which led to the sterilising apparatus. It was found that this was not so. When the door which led from the ward into the corridor was opened even a little there was no inrush of air, while there were occasional gusts out from the ward, and when the door was opened widely there was a strong outward current of air, and this although the air of the corridor was 17 degrees colder than that of the ward. Nor did it make much difference in the current of air whether the air inlets in the outer wall were open or closed. Experiments were made in every way; but even when the door and windows were closed and the air inlets open it was impossible to prevent the escape of air from the wards. As Dr. Barry says: "The inlets as frequently acted as outlets as the reverse; and the mere fact of opening the door at once caused openings which under other circumstances acted as inlets to be converted into outlets."

The next experiment was intended to test the sterilising power of the gas-jets in the air-outlet flues. To this end capsules containing a thin layer of sterilised agar-gelatine were employed. Some were placed on the floor of the ward and allowed to lie there for twenty minutes, just after a door-mat had been violently shaken in the ward. All these capsules, after being kept for three days in the laboratory, showed colonies of bacteria, varying in number with the distance at which each capsule had lain from the door-mat. Other capsules were exposed in the flue leading from the ward into the chimney into which the exit air was drawn. After exposure these capsules were kept in the laboratory under suitable conditions for a week, but no bacteria appeared in them. The capsules used in a subsequent experiment, however, showed a few colonies of bacteria even after passing the sterilising process.

In the Bagthorpe Hospital at Nottingham the results of Dr. Barry's experiments were even less satisfactory. There also the air inlets frequently acted as outlets, and as regards the sterilising apparatus Dr. Barry says:—"The arrangements made to secure the sterilisation of such air as passed through the extraction shaft were practically useless, as I found it possible to pass pieces of cotton wool and tissue paper up the shaft beyond the Bunsen gas burner without their being even singed. I was further given to understand that owing to the ease with which the gas flames of the Bunsen burner were extinguished by the draught from the smoke tower, their use had been practically discontinued."

The next experiment was made in the small-pox ward of the Bradford Corporation Fever Hospital. Here the windows were hermetically sealed, and all air was admitted by shafts which communicated with the outer air on one side, and with a chamber under the ward, where this is warmed. This warm fresh air enters the wards at the foot of each bed. The vitiated air is drawn out by openings made at the ceiling level of the ward wall over each bed. The exit air is then drawn into a powerful furnace, where some of it helps to promote the combustion, and the remainder is exposed to an estimated temperature of 800° Fahr. before being passed into the open air through the chimney. In this hospital it was found that the air inlets were wholly satisfactory; they never acted as outlets. The furnace seemed to be perfectly successful in drawing the vitiated air out of the ward, but as regards the complete sterilisation of this exit air it was not so successful. Capsules were exposed in the ward and in the chimney, as at Barnsley; but in those which were exposed to the air which was supposed to be sterilised, colonies of bacteria developed, although in far smaller numbers than in the capsules which had been exposed in the wards.

Thus no thoroughly satisfactory system of drawing out and sterilising the air of small-pox wards seems yet to have been discovered. Dr. Barry speaks with praise of the ventilating system of the Victoria Infirmary at Glasgow, of which he heard after making the observations he has recorded; but he does not seem to have made any experiments there. Few people, perhaps, realise what is involved in the attempt to sterilise the air escaping from a ward—certainly not those people who propose to do it by means of a few Argand burners. If we take a ward of twelve beds and consider that from such a ward there must escape at the very least (without allowing anything for the extra ventilation which small-pox cases require) 36,000 cubic feet of air every hour, and that to ensure complete sterilisation the whole of this air must be raised to a temperature of 212° Fahr., and if we then think of the grumbling which committees are apt to indulge in at the amount of coal required to raise this same air merely to about 65° Fahr.—the temperature of the wards—then we can make some sort of a guess at the immense amount of coal which would be required for sterilisation, and at the absurd inadequacy of the various plans which have hitherto been devised for the purpose. That it can be done no one doubts. It is a mere matter of money. Probably, however, it would cost less to wash the air than to burn it.

